# Studying the surfaces of bacteria using neutron scattering: finding new openings for antibiotics

**DOI:** 10.1042/BST20200320

**Published:** 2020-10-02

**Authors:** Nicolò Paracini, Luke A. Clifton, Jeremy H. Lakey

**Affiliations:** 1 Biofilms Research Center for Biointerfaces, Faculty of Health and Society, Malmö University, Malmö 21432, Sweden; 2 ISIS Pulsed Neutron and Muon Source, Rutherford Appleton Laboratory, Harwell Oxford, Didcot OX11 0QX, U.K.; 3 Biosciences Institute, Faculty of Medical Sciences, Newcastle University, Newcastle upon Tyne NE2 4HH, U.K.

**Keywords:** antibiotics, biological models, gram-negative bacteria, lipopolysaccharides, neutron scattering, outer membrane

## Abstract

The use of neutrons as a scattering probe to investigate biological membranes has steadily grown in the past three decades, shedding light on the structure and behaviour of this ubiquitous and fundamental biological barrier. Meanwhile, the rise of antibiotic resistance has catalysed a renewed interest in understanding the mechanisms underlying the dynamics of antibiotics interaction with the bacterial cell envelope. It is widely recognised that the key reason behind the remarkable success of Gram-negative pathogens in developing antibiotic resistance lies in the effectiveness of their outer membrane (OM) in defending the cell from antibacterial compounds. Critical to its function, the highly asymmetric lipid distribution between the inner and outer bilayer leaflets of the OM, adds an extra level of complexity to the study of this crucial defence barrier. Here we review the opportunities offered by neutron scattering techniques, in particular reflectometry, to provide structural information on the interactions of antimicrobials with *in vitro* models of the OM. The differential sensitivity of neutrons towards hydrogen and deuterium makes them a unique probe to study the structure and behaviour of asymmetric membranes. Molecular-level understanding of the interactions between antimicrobials and the Gram-negative OM provides valuable insights that can aid drug development and broaden our knowledge of this critically important biological barrier.

## Introduction

**Figure 1.**
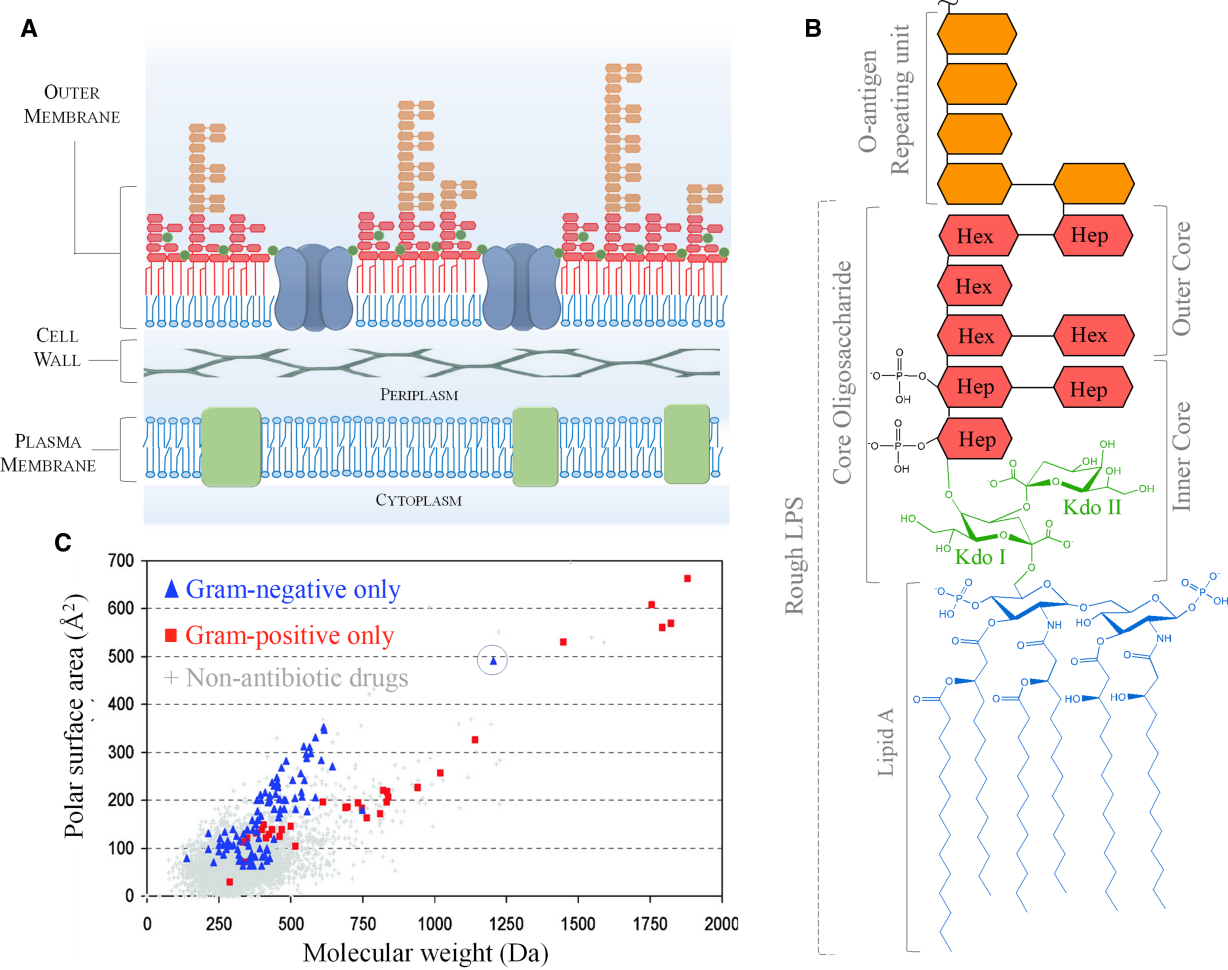
The Gram-negative cell envelope and its unique defense barrier. (**a**) Schematic representation of the three layers of the Gram-negative cell envelope. Above the plasma membrane the periplasm is 10–20 nm wide and contains the rigid peptidoglycan cell wall, which is surrounded by the asymmetric OM made of an inner PL leaflet and an outer LPS layer. LPS molecules are a mixture of short ‘rough' and longer O-antigen bearing ‘smooth' LPS and are cross-linked by divalent cations, shown as green circles. Abundant porin proteins in the OM form a molecular sieve that allows only small hydrophilic molecules across the membrane. (**b**) Basic structure of *E. coli* LPS. The structure of rough LPS is from the K-12 strain, shown without non-stoichiometric substitutions [5]. The multiple negative charges of the inner core and lipid A moieties stabilised by divalent cations, together with the six acyl chains of the lipid A, confer on the OM its unique barrier properties. Hep = heptose, Hex = hexose, Kdo = 3-deoxy-D-manno-oct-2-ulosonic acid (**c**) Physicochemical properties of antibacterial compounds. Commercial drugs are shown in a plot of polarity versus size. Antibiotics active against Gram-negative targets (blue) are not only smaller than ∼600 Da but also more polar (water soluble) than antibiotics against Gram-positive bacteria which lack the OM. The only clear outlier is PmB (circled) which avoids the porin-dictated size limitations by targeting LPS. Reprinted with permission from [11] copyright 2008 American Chemical Society.

Within the list of the highest-priority drug resistant pathogens highlighted by a recent WHO report, Gram-negative bacteria outnumber Gram-positives seven to two, with the top three critical-priority organisms all belonging to the Gram-negative class [1]. The key reason behind the success of Gram-negative bacteria in avoiding the noxious effects of antibiotics lies in the structure of their cell envelope [2]. The distinctive double membrane architecture which surrounds Gram-negative organisms comprises the canonical plasma membrane enclosed by an additional outer membrane (OM), with a thin layer of peptidoglycan sandwiched in the periplasmic space in between them (Figure 1a). The OM is the outermost layer of the bacterial Gram-negative cell and owes its unique barrier properties to the highly asymmetric composition of its lipid bilayer [3] (Figure 1a). Whilst the inner leaflet of the OM contains phospholipids (PL), the outer leaflet is made of an impermeable layer of negatively charged lipopolysaccharide (LPS) molecules, cross-linked into a tight non-covalent network by Ca^2+^ and Mg^2+^ cations. Lipid asymmetry is preserved by a specialised ensemble of proteins which ensure that LPS molecules are delivered to, and maintained within the outer bilayer leaflet, whilst misplaced PL are segregated to the periplasmic side of the bilayer or returned to the cytoplasmic membrane [4]. The unique properties of LPS arise from its tripartite structure which comprises (i) the membrane-anchoring hydrophobic lipid A moiety, consisting of a phosphorylated di-glucosamine, typically hexa-acylated, (ii) a negatively charged core oligosaccharide of 10–12 sugars units, capped by (iii) the O-antigen, an extended polymeric glycan composed of a highly variable number of repeating oligosaccharide units [5]. Whilst ‘smooth’ LPS displays all three parts, ‘rough' LPS lacks the O-antigen and can be genetically engineered to display a defined core oligosaccharide size, either complete (RaLPS) or increasingly truncated (Rb, Rc, Rd and ReLPS) (Figure 1b).

The properties of the LPS layer render the OM up to two orders of magnitude less permeable to hydrophobic molecules in comparison with canonical PL bilayers [6,7]. This leaves only small hydrophilic molecules (<600 Da) able to diffuse across the OM via non-specific transmembrane water-filled β-barrel protein pores, named porins, which are abundant in the OM and enable uptake of solutes with various degrees of substrate specificity [8]. The remarkably low permeability conferred by LPS onto the OM lipid matrix, makes the porin-mediated route the bottleneck for antibiotic entry into Gram-negative bacteria [9,10]. This is clearly exemplified by the physicochemical properties of commercial drugs active against Gram-negatives, which, with a very few exceptions, are small and hydrophilic, with a molecular mass cut-off ∼600 Da (Figure 1c). This sharp cut-off is a distinct signature of the OM molecular sieve properties, matching the maximum size of molecules allowed to diffuse through porin channels [11]. Even the most recent efforts to elucidate the rules behind molecular permeation through the OM substantially translate into rules that favour the passage of compounds through porin channels [12–14]. Crucially, LPS-imposed restriction of antibiotic entry to the porin route supports numerous other effective antibiotic resistance strategies used by Gram-negative bacteria. These include the overexpression of drug efflux pumps, mutations of critical residues in the eyelet region of the porin channels to further impede antibiotic entry and production of periplasmic enzymes, such as β-lactamases, aimed at degrading drug molecules [15]. The protective role of the OM against antibiotic penetration therefore goes beyond that of a simple physical fence and relies upon the combined roles played by proteins and lipids.

The increasingly difficult challenge of reaching targets hidden behind the OM permeability barrier has driven efforts in antibiotic research towards targeting the OM itself [16,17]. The main candidates in this area are antimicrobial peptides and peptidomimetics but, despite growing interest, this approach is still struggling to produce safe drugs for clinical use [18]. Murepavadin [19] and darobactin A [20] are amongst the latest and most promising molecules currently under development, targeting the LptD and BamA OM proteins respectively. The only clinically approved antibiotic class that directly targets LPS are polymyxins. Polymyxin B (PmB) and polymyxin E (known as colistin) bind LPS and induce OM damage leading to cell death [21]. However, due to their toxicity, their use is limited to last resort therapies for infections untreatable by any other drug [22]. Polymyxins still represent the clearest example of an antibiotic active against Gram-negative pathogens that avoids the limitations of the OM molecular sieve by directly targeting LPS. Despite their limited use however, resistance towards polymyxins is a growing concern, especially after the identification of the *mcr-1* plasmid which codes for LPS modifications that inhibit their activity [23].

A detailed understanding of the OM biophysical properties and its interactions with antibacterial molecules is therefore critical to devise novel entry routes across the first defence line of multiple life-threatening pathogens. However, obtaining high-resolution structural information about the OM directly from the surface of bacteria is a challenging task. An alternative approach to the problem consists of reconstituting OM components *in vitro* to create models that mimic the OM structure and composition. These enable structural analysis using techniques such as scattering, under controlled conditions [24]. In the last ten years, a growing body of work based on *in vitro* models of the OM has provided a new platform for the investigation of the properties and behaviour of this crucial biological barrier. In particular, the use of specular neutron reflectometry (NR) offered valuable insights into the structure–function relationship of different LPS chemotypes and the effects of antimicrobials interacting with OM models [25–29]. Here we review the contribution that neutron scattering has provided to the investigation of *in vitro* models of the OM and their interaction with antimicrobial compounds. We outline the advantages of neutron scattering in the context of structural techniques amenable to study the interaction of antibiotics with the bacterial surface, together with the current limitations that this approach entails.

## Approaches to study the direct effects of antibiotics on the bacterial cell envelope

### Methods to investigate antibiotics effect on the native bacterial surface

Structural investigations of the OM began with the introduction in the late 1950's of electron microscopy imaging of intact and sectioned bacteria which produced the first visual evidence of the double membrane envelope of Gram-negative organisms [30]. Since then, electron microscopy has contributed to the analysis of the large-scale morphological alterations of the bacterial envelope caused by clinically relevant antibiotics, as extensively reviewed recently by Cushnie et al. [31]. Negative-stain electron microscopy held a pivotal role in the early characterisation of the Gram-negative cell envelope morphology and still represents an accessible way to image whole bacteria. However insufficient resolution and invasive staining procedures have limited its development into a technique amenable to obtain detailed structural information on the OM. Recently, important progress has been made in the field of electron cryotomography, which has been used to resolve the structure of large membrane protein complexes directly within the native cell envelope of intact organisms [32,33] including the multidrug efﬂux pump AcrAB-TolC [34] involved in several critical antibiotic resistance mechanisms in Gram-negative bacteria [35].

The advent of atomic force microscopy (AFM) paved the way for the investigation of the properties and behaviour of bacterial surfaces under conditions that are much closer to those relevant to antibiotic action *in vivo* [36]. AFM has demonstrated the potential to monitor real-time effects of antimicrobials on the surfaces of living bacterial cells under native conditions [37,38] and can achieve sub-second time resolution, enabling single molecule tracking on the bacterial surface [39]. Additionally, antibiotic-modified AFM tips have shown the potential to investigate the forces and dynamics characterising the interactions between antimicrobials and their surface exposed ligands [40]. To date, some of the highest resolution images of the OM, detailing the structure of the mesh-like molecular sieve created by arrays of porins, have been obtained using AFM [39,41,42]. AFM has shown tremendous potential to provide molecularly detailed images of the bacterial surface but doing so under physiological conditions remains technically challenging and the highest resolution images are usually collected on purified OM fragments.

Often used in combination with AFM, fluorescence microscopy has been extensively used to investigate the bacterial surface and its interaction with antibiotic molecules [43]. Fluorescent labels attached either to components of the cell surface, such as PL [44] and LPS [41,45] or directly to the antibiotic molecule [46], enable monitoring of drug permeation and distribution, together with membrane dynamics. Fluorescently labelling antibiotics however requires covalent modification of the molecular structure, which in most cases alters the properties of the drug. Nevertheless, fluorescence microscopy remains one of the most effective ways to investigate the lipid component of the OM on the natural bacterial surface [41].

### Modelling the OM *in vitro*

**Figure 2.**
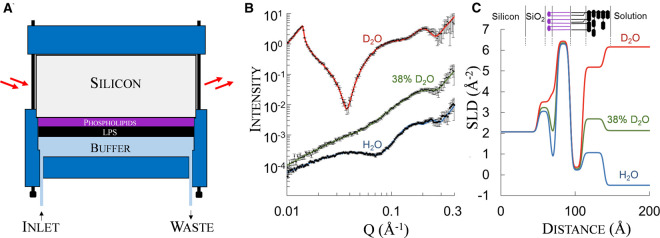
Neutron reflectometry analysis of asymmetric OM model. (**a**) Sample holder for neutron reflectometry experiments. The neutron beam passes through a thick silicon crystal and is reflected at the solid/liquid interface. Here, the supported asymmetric OM model, containing deuterated PL and hydrogenous rough LPS is surrounded by a buffer solution that can be easily exchanged through the cell inlets and outlets. (**b**) Data obtained from a typical NR experiment. The intensity of the specularly reflected neutron beam is measured as a function of Q, a combination of neutron wavelength and incident angle [53]. The asymmetric PL/LPS OM model is measured at three solution isotopic conditions (data points) which are simultaneously fitted (lines) to a common model that describes the interfacial bilayer structure shown in **c**. Curves are offset for clarity and the intensity is multiplied by Q^4^ to correct for the inherent decline in intensity with increasing angle. (**c**) structure of the deuterated PL (purple)/hydrogenous LPS (black) asymmetric bilayer described by the scattering length density (SLD) distribution of nuclei across the membrane profile. Note how SLD (in effect the neutron refractive index) of the acyl chains differs between natural LPS, deuterated lipids and different concentrations of D_2_O. The corresponding structure of the interface is shown above the profile. **b** and **c** are reproduced from [26].

The technical challenges posed by the complexity of natural cell membranes are often overcome by accepting the compromise of performing biophysical experiments on *in vitro* model membranes reconstituted using synthetic or purified membrane components. The appeal and convenience of model membranes is not only limited to their reduced complexity but extends to their ease of preparation and versatility. Under the right conditions, lipids self-assemble into vesicles amenable for solution studies which can later be fused onto hydrophilic surfaces to form planar lipid bilayers suitable to be investigated by several biophysical approaches [47]. Although some degree of lipid asymmetry can be achieved [48], vesicle fusion do not generally yield asymmetric lipid bilayers that reproduce the OM lipid distribution. Notably, a novel approach to create *in vitro* OM model bilayers via vesicle fusion has been proposed by Hsia et al. [49] who fused purified OM vesicles onto a polymer functionalised surface showing that the resulting planar bilayer largely retained the native protein orientation. The most effective strategy to impose lipid asymmetry onto planar lipid bilayers is to use Langmuir–Blodgett and Schaefer techniques to sequentially transfer lipid monolayers from the air/water interface onto solid substrate [50]. This method provides full control over the lipid composition of the bilayer leaflets and can be used to sequentially stack PL and LPS to yield a highly asymmetric *in vitro* model of the OM [51] (Figure 2).

Asymmetric PL/LPS planar bilayers represent a unique platform for structural studies of molecular interactions of a wide range of solutes, including small molecule antibiotics and antimicrobial peptides, with a simplified mimic of the Gram-negative surface [25–29,52]. Such models enable structural characterisation of highly asymmetric bilayers in an aqueous environment, with precise control over temperature, pH and buffer composition, at a resolution unattainable on the natural OM (Figure 2) [24]. The flat nature of planar model membranes allows precise determination, using reflectometry techniques, of the molecular structure of the bilayer along the axis perpendicular to the surface [53]. In particular, the use of neutrons as scattering probes for specular reflectometry experiments, provides some important advantages over the more widespread and often complementary X-ray scattering approaches. The key difference between X-ray and neutron scattering lies in the different interactions these two probes display with matter. Whilst X-rays (i.e. photons) are deflected by the electron cloud surrounding molecules, neutrons are mainly scattered by the atomic nuclei within. This has three major consequences which constitute the core advantages of neutron scattering in a biological context: (i) sensitivity to light elements (ii) differential sensitivity to isotopes, particularly hydrogen and deuterium and (iii) high penetration depth through complex sample holders [54]. In particular, the large difference in the neutron scattering magnitude of hydrogen and deuterium enables the exploitation of selective deuteration of PL and LPS to create isotopically asymmetric OM models, providing the contrast necessary to distinguish the individual structure of the two leaflets in the OM model bilayers [55]. The contrast between the sample and the aqueous solution background can be further adjusted by mixing H_2_O with heavy water (D_2_O) in the buffer bathing the bilayer. This enables measurement of multiple sets of reflectivity data at different aqueous isotopic compositions, which can later be simultaneously fit to a single model of the interface (Figure 2b). The data obtained through the analysis of NR curves provides a description of the distribution of atomic nuclei across the 5–10 nm thin space spanned by the lipid bilayer. Different parts of the OM model, such as PL and LPS head group and tail regions, can thus be characterised in terms of their individual thickness, elemental composition and water content in an aqueous environment (Figure 2c). For an in-depth description of neutron scattering and its application to study planar asymmetric bilayers the reader is referred to the recent book chapter by Clifton et al. [53] which details theoretical and practical aspects of the experimental set up.

## Studying effects of antimicrobials on OM models of increasing complexity

**Figure 3.**
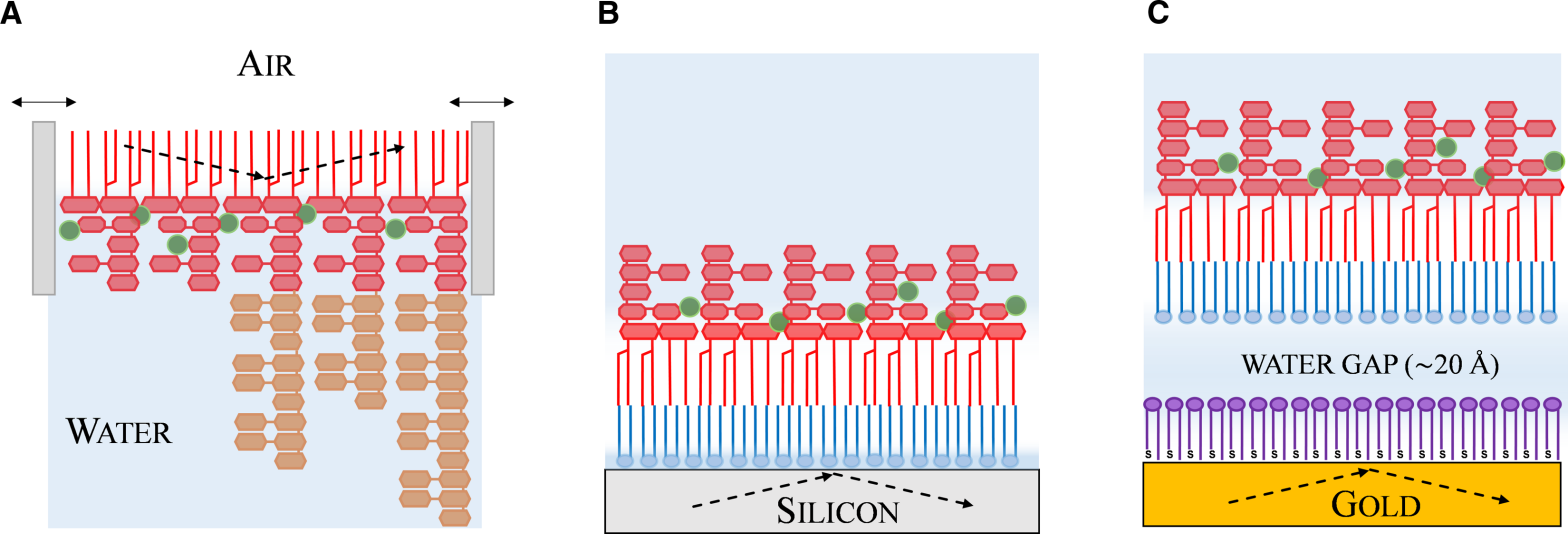
OM models for structural studies of antibiotic interactions. (**a**) LPS Langmuir monolayer at the air/water interface enclosed by the movable barriers of a Langmuir trough (**b**) solid supported asymmetric PL/LPS bilayer (**c**) floating PL/LPS bilayer. The gold surface is coated with a monolayer of thiolated lipids (purple) which creates a ∼20 Å water cushion between the substrate and the asymmetric model membrane. Dashed lines represent the path of the beam in a NR experiment.

In the last ten years, the combination of NR and *in vitro* OM models has shed a new light on this ever more relevant biological barrier. Here we review the contribution of NR to the study of antimicrobial interactions with three types of OM models of increasing complexity: (i) LPS monolayers at the air/water interface, (ii) solid supported asymmetric bilayers and (iii) floating asymmetric bilayers (Figure 3).

### LPS Langmuir monolayers

As well as constituting the starting point for the assembly of asymmetric PL/LPS bilayers, Langmuir monolayers of LPS, spread at the air/water interface, provide the simplest mimic of the outer leaflet of the OM (Figure 3a). NR has been employed in combination with X-ray reflectometry to study the structure of both rough [56] and smooth [57] LPS monolayers at the air/water interface. This allowed the determination of the spatial arrangement of the lipid A and core oligosaccharide regions as well as the conformation of the O-antigen polymeric chains in relation to surface pressure and divalent cation concentration. Although NR has contributed to the characterisations of these systems, to our knowledge, only one study to date has addressed LPS-antibiotic interaction using NR at the air/water interface [58]. The majority of structural studies used X-rays to analyse the effects of several antimicrobials including novobiocin [59], LL37 [60], plasticins [61] protegrin [62] and fish protamine [63,64] together with the effects of surface pressure and divalent cations [52,65,66] on LPS monolayers. Notably, OM proteins such as the OmpF porin can also be incorporated in Langmuir monolayers and NR has been used to study the insertion of the antimicrobial protein colicin N (ColN) into OmpF-rich PL monolayers at the air/water interface [67].

### Supported PL/LPS asymmetric bilayers

Sequential Langmuir–Blodgett and Schaefer depositions onto a solid substrate of monolayers of deuterium labelled PL and hydrogenous LPS yield isotopically asymmetric PL/LPS bilayers (Figure 3b). Unlike the case of the air/water interface, in order to probe these buried solid/liquid interfaces the significantly higher penetration depth of neutrons over X-rays is an essential feature. Isotopically asymmetric OM models containing either the basic lipid A structure or rough LPS with a complete (Ra) or truncated (Rc) core oligosaccharide were first shown to retain high levels of lipid asymmetry and coverage upon deposition on silicon substrates in a 2013 study by Clifton et al. [68]. A follow up investigation combined the monolayer and the PL/LPS bilayer approaches to characterise the loss of structural integrity caused by divalent cation removal on the OM models [52]. Building on this work, the first application of NR and asymmetric PL/LPS bilayers for antimicrobial studies elucidated the effects of LPS core oligosaccharide size on the electrostatic binding of the antimicrobial protein ColN [27]. The study elegantly demonstrated the ability of the uncharged outer core oligosaccharide of LPS to screen electrostatic binding of the cationic antimicrobial to the anionic LPS inner core. The structural data enabled the quantification of the amount of ColN adsorbed on the membrane as a function of the ionic strength of the buffer solution as well as the bound orientation of the antibacterial protein. In a separate study, a different group combined AFM and NR to investigate the effects of two types of plasticins on asymmetric OM models, highlighting once again the importance of the cationic nature of these type of antimicrobial peptides for their interaction with the OM [69]. More recently, Vandera et al. [70] implemented, for the first time, the use of PL/LPS bilayers to address the fusion mechanism of a bacterial drug delivery system with its model OM target. Alongside with complementary microbiological assays, NR provided novel structural insights into the initial stages of translocation of a fluidosome-based drug carrier across the Gram-negative surface.

Due to their unique interaction with the OM, antibiotics of the polymyxin family have been the subject of several studies on *in vitro* OM models [26,28,29]. Han et al. [28] used asymmetric PL/lipid A bilayers to investigate the effects of PmB along with two novel synthetic polymyxin analogues in relation to lipid A acylation patterns. In another comparative study, the same group addressed the differences in the mode of action of PmB and the related antimicrobial octapeptin A3, focusing on the role of lipid A charge modifications which play a primary role in bacterial resistance towards polymyxins. Unlike PmB, octapeptin A3 was shown to retain the ability to penetrate the OM model containing modified lipid A, in agreement with its greater *in vivo* bactericidal activity against PmB-resistant Gram-negative pathogens [29]. The latest NR study on the effects of PmB on OM models shed light on the molecular basis of its long-known temperature dependent bactericidal activity [26]. Using a combination of NR and infrared spectroscopy the disruptive effects of PmB were shown to be strongly dependent on the transition of the LPS leaflet from a gel to a liquid crystalline phase, demonstrating the previously disputed biological relevance of LPS phase transition in the OM.

### Floating PL/LPS asymmetric bilayers

Combining a functionalised gold substrate with the asymmetric model described above enables a third type of model with the PL/LPS bilayer floating on a ∼20 Å thick water cushion separating it from the solid interface (Figure 3c). Building on the successful formation of asymmetric silicon supported OM models, Clifton et al. implemented the approach developed by Hughes et al. [71,72] to deposit asymmetric bilayers on top of a self-assembled monolayer of ω-thiolipids chemically grafted onto a gold substrate [25]. The structure of the floating PL/LPS bilayer was determined under multiple conditions using polarised NR and magnetic contrast, a type of NR measurement devised to further reduce uncertainty in the interpretation of the scattering data from highly complex interfacial structures [73]. The introduction of the ω-thiolipid layer enabled the formation of a ∼20 Å thick water reservoir separating the PL head groups from the substrate, which separated the OM model from the solid support while still retaining high levels of lipid asymmetry. The model was used to test the effects of calcium removal and the antimicrobial proteins lysozyme and lactoferrin. Both antimicrobial proteins tested were shown to interact with the bilayer and, in line with that observed on the natural OM, lactoferrin led to a more substantial disruption of the OM model structure.

## Outlook

Access to neutrons remains limited to large scale facilities, however, thanks to their distinctive properties, their use to study biological systems, provides unique access to structural information, both *in vitro* and, more recently, *in vivo* [74,75]. Here we focused on the contribution of NR to the analysis of asymmetric membrane models at the solid/liquid interface, which is a clear example of a case of study in which no other structural techniques could be employed. The combination of NR and *in vitro* OM models has shown a clear potential for the investigation of LPS and the asymmetric OM structure [52,56,57,68], its interaction with antimicrobials [25–29] and drug delivery systems [70]. The majority of the present studies have focused on rough LPS species due to their well-defined structures but the recent work by the group of Schneck et al. has demonstrated the possibility to assemble stable smooth LPS layers both at the air/water [57] and at the solid/liquid interface [76]. Work to incorporate smooth LPS on both supported and floating bilayers is currently in progress and will soon provide a platform to investigate artificial bacterial surfaces closer to that of wild-type and pathogenic bacteria. As well as mediating PmB resistance, LPS modifications like those encoded by the *mcr-1* plasmid, have recently been shown to render the OM more susceptible to hydrophobic antibiotics and osmotic shock [77]. Thus, further development of OM models containing resistant LPS [29] could help clarify the molecular basis behind the multifaceted effects of LPS modifications involved in antibiotic resistance [4]. The interaction between LPS and OM proteins plays a critical role in OM stability and is at the basis of its effective barrier properties [3,78]. Building on the current approaches used to reconstitute porins *in vitro* [49,73], the development of an OM model containing oriented porin channels within a PL/LPS asymmetric bilayer suitable for structural analysis would represent an important step forward towards models that enable investigation of the porin-LPS interplay. Furthermore, there is evidence suggesting that porins have a tendency to form large ordered domains within the OM [42]. Reconstituting porins in a planar PL/LPS bilayer would enable the study of the in-plane organisation of the poorly characterised porin-rich OM regions under native-like conditions using grazing incidence small angle scattering and diffraction techniques [79]. Finally, the development and implementation of complementary techniques to study planar asymmetric bilayers is essential to provide details on dynamical aspects and in-plane information that cannot be accessed by NR [47]. Given its isotopic sensitivity, infrared spectroscopy is amongst the best suited candidates to supplement NR data and has already been shown to provide highly complementary information on asymmetric OM models [26]. These are just some of the directions in which the methods described here could be taken in the future. What is certain, is that as the model membrane toolbox expands, so will the number of questions these approaches will contribute to answer.

## Perspectives

- Neutron reflection is a powerful tool to study the structure of lipid bilayers and unique in its ability to reveal the asymmetric distribution of molecules across membranes. Lipid asymmetry is a crucial feature of the Gram-negative bacterial outer membrane, an important biological barrier that plays a fundamental role in the development of antibiotic resistance by some of the most clinically relevant pathogenic bacteria.
- The combination of neutron reflectometry and *in vitro* asymmetric bilayers, that mimic the structure of the outer membrane, is progressively gaining traction as a tool for the structural characterisation of antibiotic-membrane interactions.
- The development of increasingly realistic outer membrane models, together with further advance and implementation of techniques complementary to NR, has the potential to probe aspects of membrane biology inaccessible by other methods.

## Abbreviations

AFM: atomic force microscopy
LPS: lipopolysaccharides
NR: neutron reflectometry
OM: outer membrane
PL: phospholipids
PmB: polymyxin B
SLD: scattering length density
